# The Present and Future of Zone 0 Endovascular Arch Reconstruction

**DOI:** 10.3390/jcdd13020093

**Published:** 2026-02-13

**Authors:** Ming Hao Guo, Robert-James Doonan, Mark Rockley

**Affiliations:** 1 Division of Cardiac Surgery, University of Ottawa Heart Institute, Ottawa, ON K1Y 4W7, Canada; 2 Division of Vascular Surgery, McGill University, Montreal, QC H4A 3J1, Canada; 3 Division of Vascular Surgery, University of Calgary, Calgary, AB T2N 1N4, Canada

**Keywords:** aortic arch, endovascular, open surgery

## Abstract

Thoracic aortic pathology involving the aortic arch is most commonly treated with open total arch replacement. However, open surgery is still associated with significant risk of mortality and morbidity, particularly in the elderly, patients with high-risk comorbidities, and those with previous cardiac surgery. Multiple endovascular approaches to enable zone 0 arch reconstruction have been developed, including custom-made, physician-modified, and off-the-shelf fenestrated/branched endografts. The initial experiences of this approach have been plagued by high incidence of stroke; although improvements have been made over the past decade, it remains suboptimal. Several factors contribute to this stagnation, including limited descriptive studies with small sample sizes, heterogeneous patient populations, varied techniques, and lack of data granularity and standardization. These limitations reduce the ability to analyze factors that could improve patient selection, device design, and procedural techniques. In addition, consistent follow-ups have not been reported, and the long-term outcome of these interventions are unknown. To address these issues, a randomized controlled trial of open versus endovascular arch repair or multicenter registry with standardized data reporting, follow-up protocol, and sufficient sample size would be needed. High-quality data will help identify patient clinical or anatomical features as well as procedural factors that can improve outcomes.

## 1. Introduction

Pathology involving the proximal aortic arch, including aneurysm, dissection, penetrating aortic ulcer, intramural hematoma, or pseudoaneurysm are most commonly treated with open total arch replacement. Given its anatomical proximity to the innominate artery (zone 0), left carotid artery (zone 1), and left subclavian artery (zone 2), surgery of the aortic arch requires cardiopulmonary bypass, hypothermia, and circulatory arrest for reconstruction of the cerebral vessels. Despite advancement and innovation in surgical techniques, open surgery can still be associated with elevated risk of mortality and morbidities, particularly in elderly patients, those with previous cardiac surgical interventions, and patients with significant comorbidities or pre-operative functional limitations.

Initial work on arch vessel incorporation into endovascular devices began in the late 1990s. However, endovascular total arch repair in zone 0 only started to gain popularity and emerged as an alternative to open surgical repair in the last 10–15 years. Initially, physician back-table modification of existing thoracic endovascular grafts, parallel endografting with chimney or snorkel techniques, and in situ fenestration with laser or needle were used to restore blood flow to cerebral vessels. Since the first description of the modular branched device by Chuter et al. in 2003, significant work has been conducted in developing custom-made devices (CMDs) and off-the-shelf fenestrated and branched endografts [1]. Endovascular repair promises a minimally invasive approach, avoiding morbidities associated with cardiopulmonary bypass and hypothermia, and provides much faster postoperative mobilization and recovery. However, it is also associated with unique challenges, and despite more than two decades of experience, endovascular total arch repair has not gained widespread use when compared to other transcatheter interventions developed during the similar period.

In this review, we will evaluate the current state and discuss the future prospects of zone 0 endovascular intervention.

## 2. Present State

Multiple approaches are currently available to enable endovascular total arch repair, including CMDs, off-the-shelf devices, physician-modified endografts, parallel grafts, and in situ fenestration.

### 2.1. Branch Devices

#### 2.1.1. Off-the-Shelf Devices

The thoracic branch endoprosthesis (TBE; W. L. Gore & Associates, Inc., Flagstaff, AZ, United States is an off-the-shelf, single retrograde branch device initially designed for single-stage zone 2 repair with left subclavian artery (LSA) revascularization [2]. Investigators have extended its use in zone 0 arch reconstruction with extra-anatomical bypass of the left carotid and left subclavian arteries, and extension of the single branch in the innominate artery [3]. Dake et al. reported on eight patients with zone 0 TBE in patients with aneurysm pathology only with a combination of extra-anatomical bypass; one patient experienced major stroke, while three patients developed endoleak on follow-up. The innominate branch remained patent at 12 months in 100% of patients [3]. A midterm analysis of the same study with 3 years of follow-up found no device migration, fracture, or innominate branch loss, and no new endoleaks [4].

The NEXUS Aortic Arch Stent Graft System (Artivion, Kennesaw, GA, United States) is also an off-the-shelf, single-branch device, specifically designed for zone 0 deployment with adjunctive extra-anatomical bypass of the left carotid and left subclavian arteries [5]. In contrast to TBE, the innominate module enables antegrade flow into the innominate artery. The initial experience for patients with mixed aortic pathology reported a 30-day mortality of 7.1%, stroke of 3.6% (all of which were non-disabling), and a combined mortality and stroke rate of 10.7%. Two additional cerebrovascular events were reported at 1 year, with device-related unplanned reintervention of 10.7% at 1 year and 29% at 3 years [6].

There are several other single-branch devices on the market, including Castor Microport. However, experiences with these grafts in zone 0 are limited. There are currently no off-the-shelf multibranch devices.

#### 2.1.2. Custom-Made Devices

The Relay Branch System (Terumo Aortic, Sunrise, FL, USA) is a custom-made, single- or multibranched aortic arch endograft designed for zone 0 deployment [7]. Initially designed for single or double branches, it has a wide window in the superior aspect of the endograft to accommodate these antegrade branches, usually for the innominate artery and left carotid artery. Additionally, a third retrograde branch can be incorporated for the subclavian artery. Initial experience included 43 patients with mixed aortic pathology from 10 centers, with an in-hospital mortality of 9%, disabling stroke of 7%, and non-disabling stroke of 19%. At median follow-up of 16 ± 18 months, 23% of patients died [8]. Recently, an international experience with the Relay graft reported a disabling stroke of 4.1% and non-disabling stroke of 5.4% [9].

The Cook a-Branch endograft (Cook Medical, Brisbane, Queensland, Australia) is a custom-made, multibranched aortic arch endograft designed for zone 0 deployment [10,11]. It can be designed with two or three large internal branches in antegrade or retrograde configuration and can include one pre-loaded catheter to facilitate cannulation. No surgical debranching is required in the 3-branch configuration. Recently, a multicenter study of 213 patients with mixed aortic pathology who underwent zone 0 a-Branch reported an in-hospital mortality of 8.9%; patients with non-dissection pathology had more postoperative strokes compared to patients treated for dissection (17.7% vs. 4.5%; *p* < 0.01) [12]. On multivariable analysis, previous type A aortic dissection was protective for the composite outcome (odds ratio, 0.2; 95% confidence interval, 0.1–0.6), whereas degenerative aneurysm with involvement of zone 0 or 1 was predictive of postoperative stroke (odds ratio, 3.7; 95% confidence interval, 1.2–11.8). Nana et al. similarly noted in their study that native aorta zone 0 landing zones were associated with a 13.5% risk of stroke [13].

Outcomes for single- or multibranch devices are summarized in Table 1.

### 2.2. Fenestrated Devices

#### 2.2.1. Physician-Modified Fenestrated Devices

Zone 0 physician-modified endografts (PMEG) involves back-table creation of fenestrations for aortic arch vessels and can also include the use of in situ fenestration. In the literature, stroke occurred in 7–10% of patients, and type 1 endoleak in 10–15% of patients, some of whom required reintervention during follow-up [27,28]. The use of bridging stent for the innominate and left carotid artery in zone 0 PMEG is not always performed, which decreases the need for wire manipulation in the cerebral vessels. Bacri et al. reported 74 patients with mixed aortic pathology who underwent double fenestrated PMEG without bridging stents with excellent outcomes [29]. This technique used a small fenestration for the subclavian and a large fenestration or scallop to include both the innominate and left carotid artery. The subclavian fenestration is pre-loaded with a wire which is snared for through-and-through access, and tension on the through-and-through wire during deployment helped to align the fenestrations, reducing malrotation and graft manipulation. In a cohort where 27% of cases were emergent, 30-day mortality was 1% and stroke was 5%; only 1 patient had early type 1a endoleak, which was successfully treated [29]. However, fenestrated grafts can be technically challenging as alignment must be perfect. In addition, long-term follow-up is required to understand its impact on aneurysm exclusion and potential endograft malposition over time, particularly if no bridging stents were used.

#### 2.2.2. Custom-Made Fenestrated Devices

The fenestrated CMDs aim to maintain patency of the cerebral vessels through fenestrations or scallops without bridging stents. The Relay stent graft (Terumo Aortic, Sunrise, FL, USA) can also be customized to include a customized scallop, fenestration, or a fenestration and a scallop. The delivery system consists of a pre-curved catheter with a proximal capture mechanism. Adjunctive surgical debranching is often required. Sica et al. reported 49 patients with mixed aortic pathology, 25 of whom required proximal landing in zone 0 [30]. Two patients experienced non-disabling stroke, and no type 1 endoleak was observed at 1 month.

A fenestrated arch CMD is also available from Cook Medical (Bloomington, IN, USA), featuring varying combination of fenestrations and scallops, targeting one or two of the cerebral vessels, with adjunctive surgical debranching if required. Alignment of fenestrations are facilitated by a spiral wire attachment of the endograft to a pre-curved delivery system, and an inner catheter for establishment of a through-and-through wire [31]. Tsilimparis et al. reported 12 cases with zone 0 coverage and mixed aortic pathology; 1 patient died from retrograde type A dissection, and 1 patient had a stroke.

The Najuta endograft (Kawasumi Laboratories, Inc., Tokyo, Japan) is a custom fenestrated stent graft covered in polytetrafluoroethylene with single or multiple unsupported fenestrations. Unique to the Najuta graft, the stent framework itself can be customized to conform to patient-specific aortic morphology [32]. Isernia et al. reported results of 76 patients with mixed aortic pathology; of the 70 patients who had zone 0 coverage, 1 patient died peri-operatively, 3 patients had a stroke, and 2 patients had type 1 endoleak from graft migration [33].

Comparison between branched and fenestrated arch endografts is limited by small sample size in both groups. In one study of 29 patients, Tsilimparis et al. reported 20% 30-day mortality and 14% major stroke for fenestrated grafts, and 0% mortality and 7% stroke for branched grafts; however, the difference did not reach statistical significance [23]. On the other hand, in another study of 54 patients comparing the Relay branch arch endograft to the Najuta fenestrated device, peri-operative mortality was 10% and 0%, and the rate of major stroke was 10% and 3%, respectively [34]. Ultimately, in a recent systematic review and meta-analysis, no difference was noted between fenestrated and branched grafts in terms of mortality and stroke, and fenestrated grafts had higher incidence of endoleak (9.8% vs. 2.6%; *p* = 0.03) [35].

It should be noted, however, that fenestrated and branched devices are not necessarily interchangeable in a given patient. A fenestrated graft requires apposition in the aortic arch and therefore, large aortic arches are not appropriate. Consequently, fenestrated techniques are more suitable for distal arch and proximal descending thoracic aneurysmal pathologies where the arch can be considered as part of the seal zone. Branched devices can be used in larger aortic arches and applied to more proximal arch aneurysmal disease. Given rotational deployment concerns for fenestrations, CMD branch devices are generally preferred when possible.

### 2.3. Parallel Stent Grafts

Zone 0 endograft with parallel stents (chimney or periscope) for maintenance of cerebral blood flow has also been used. The technique does not require modification of off-the-shelf devices; however, there are significant concerns regarding type 1a endoleak from the interface between the parallel stent and the main body endograft, known as gutter leak. In addition, the long-term results of parallel grafts remains discouraging, with a reported rate of aneurysm-related mortality of up to 42.3% in 5 years and freedom from reintervention in 47.2% of the cases in 5 years [36,37]. In a study comparing branched endograft to chimney technique, chimney technique had significantly higher incidence of type 1a endoleak (33.3% vs. 0%; *p* < 0.01), and lower aortic event-free rate at 8 years of follow-up [22]. Therefore, parallel stent grafts are now mostly reserved for emergency cases or as a bail-out strategy.

## 3. Current Limitations of Zone 0 Endovascular Arch Repair

### 3.1. Stroke

As worldwide experiences in zone 0 endovascular arch repair have grown, the incidence of stroke cannot be understated. In a systematic review of zone 0 TEVAR (thoracic endovascular aortic repair), the pooled incidence of cerebrovascular events was 9.5% [38]. Unlike open arch replacement, where cerebrovascular events could be influenced by cerebral malperfusion, degree of hypothermia, embolism, or anticoagulation, stroke in endovascular arch repair is most commonly caused by embolic events from atherosclerotic debris, which is influenced by the patient’s atheroma burden and the degree of wire manipulation required within the aortic arch. It is known that a more proximal landing zone increases risk of stroke; however, even amongst patients who received zone 0 endovascular arch repair, those with degenerative aneurysm that involved zones 0 or 1 were more likely to experience clinically significant stroke than those with degenerative aneurysm limited or distal to zone 2 [10]. In the same study, over 90% of strokes had an ischemic component, further supporting that most strokes are caused by embolic events or hypoperfusion.

Current best practices in stroke prevention for endovascular arch intervention were outlined by Cao et al., including pre-operative optimization of anemia, adequate anticoagulation, the use of CO_2_ and high-volume saline flush for the endograft, minimizing unnecessary wire exchanges within the arch, clamping and retrograde flushing of carotid arteries during and after endograft deployment, and maintenance of intra-operative and postoperative blood pressure and replacement of blood loss [39]. However, it is unclear whether these maneuvers have indeed decreased the risk of postoperative stroke. To date, patient selection may be the most important element in stroke prevention; however, systematic risk stratification of arch atherosclerosis burden and its relationship with the incidence of stroke in endovascular arch repair have not been reported.

In the Transcatheter Aortic Valve Implantation (TAVI) literature, cerebral protection devices demonstrated potential for decreasing the incidence of disabling stroke [40]. Although there are debates on their clinical efficacy, some of these devices are approved and used in the United States. However, the disadvantages of these current devices include difficult deployment in tortuous cerebral vessels and interference with deployment aortic and branch endografts. In the future, a cerebral protection device that is suitable for endovascular arch procedures may be designed and considered in selected patients if there is high burden of intraluminal disease.

The long-term patency of bridging stents in the cerebral vessels and potential risk of stroke on follow-up is also poorly understood. In a recent study of 39 patients who underwent arch branch devices, branch thrombus was observed in 10% of the branches, most commonly within the innominate artery branch and none in the LSA branch. Symptoms associated with branch thrombus were observed in five out of the seven patients, including upper limb ischemia, stroke, or cerebral hypoperfusion [41]. The roles of antiplatelet and anticoagulation therapy in peri-operative and late stroke after endovascular arch repair are also unclear, as they are not always prescribed in the postoperative period and on discharge [42]. However, this may be considered as patients who receive carotid stenting are routinely managed with antiplatelet therapy. The US aortic research consortium found that visceral target vessel patency was improved in patients who were prescribed dual antiplatelet therapy postoperatively [42]. On the other hand, the risk of haemorrhagic stroke under dual antiplatelet therapy should also be considered in this patient population.

### 3.2. Endoleak

Proximal and branch seal are sometimes difficult to obtain due to arch angulation and branch vessel pathology. Type 1a endoleak is particularly difficult to manage in zone 0 endovascular arch repair, as options for proximal extension are often limited. Fortunately, type 1a endoleak is uncommon, particularly for patients who had previous ascending aortic replacement, where a long, straight prosthetic graft serves as an excellent landing zone that minimizes proximal endoleak. In patients with dissection extension into the cerebral vessels, type 1c endoleak from the false lumen may be anticipated, and embolization of the false lumen is often required. A standardized approach has previously been described involving staged supra-aortic artery debranching to create an adequate landing zone prior to zone 0 arch repair if indicated [43]. In addition, nearly half of all early endoleaks resolve or improve on subsequent imaging without intervention, indicating that it may be reasonable to observe and reassess endoleak with imaging [10]. Nonetheless, endoleak in zone 0 endovascular arch repair and its long-term ramifications have not been studied.

### 3.3. Flow Dynamic and Cardiac Remodeling

The design of retrograde branch allows for cannulation and branch stent placement via a transfemoral approach; however, blood flow in the retrograde branches is not physiologic, and whether this abnormal flow pattern may impact cerebral function, particularly in the long-term, is currently unknown. Flow simulation experiments comparing antegrade to retrograde branches in the renal arteries have shown similar flow rate between antegrade and retrograde branches if the bridging stent is short; if the bridging stent is long (>200 mm), however, then the flow rate from the retrograde branch is decreased [44]. Similarly, for patients who received TBE with a single retrograde branch in the LSA, no difference was observed in peak LSA pressure and flow rate before and after the procedure [45]. Interestingly, in a simulation study for 3-branch devices, by changing the LSA branch from retrograde to antegrade configuration, although the volume flow rate in the LSA is increased by almost 80%, a decrease in volume flow rate in the antegrade innominate artery of approximately 25% was observed [46]. This suggests that the most optimal configuration for a 3-branch device may, in fact, involve a combination of antegrade and retrograde branches. Optimal configurations may also be different in smaller aortic arches where total antegrade or retrograde inner branches may result in lumen crowding.

Aortic stent graft in the thoracoabdominal aorta has also been shown to artificially increase aortic stiffness. In a systematic review, aortic stiffness as measured by pulse–wave velocity was increased in patients who underwent EVAR (endovascular aortic repair) and TEVAR, which was not observed for patients who underwent open surgical repair [47]. Aortic stiffness has been associated with impaired coronary blood flow and myocardial perfusion, causing myocardial fibrosis, left ventricular hypertrophy, and myocardial dysfunction [48]. This is particularly important in zone 0 endovascular arch repair, as the stent graft is moved further proximally into the ascending aorta, potentially increasing cardiac stress. Recently, a single-center study looking at patients with Marfan syndrome reported increased risk of future type B dissection for those who had received surgical aortic root replacement compared to those who did not, suggesting a possible relationship with the use of non-distensible, rigid material in the ascending aorta. The impact of stent graft in the ascending aorta on cardiac function and distal aortic disease needs to be studied further.

### 3.4. Long-Term Outcomes

Ultimately, the most important question that remained unanswered is the long-term clinical and imaging outcomes of patients who underwent zone 0 endovascular arch repair. Long-term outcomes can be influenced by many factors, many of them discussed above, including peri-procedural complications, branch patency, stent migration, occurrence of early and late endoleak, and cardiac stress over time, all of which could lead to the need for aortic reintervention, aortic-related and non-aortic-related mortality. The importance of studying long-term outcomes is further exemplified by the EVAR literature, where although EVAR had been demonstrated to have early lower periprocedural mortality and morbidity compared to open surgical repair, by 8 years follow-up, the survival benefit had eroded [49]. For patients who underwent fenestrated EVAR, aneurysm sac regression is associated with significant survival advantage compared to those with failure to regress [50]. It is critical to understand the long-term outcomes of endovascular arch interventions to optimize patient selection, technique, and decision making.

### 3.5. Open Surgery as the Benchmark

The outcomes of open total arch replacement have improved significantly over the past decade, with the development and incorporation of neuroprotective, total body perfusion and temperature management strategies. Some centers have been able to produce excellent outcomes; most recently, Ram et al. reported an operative mortality of 0.4% for first-time total arch replacement and 3.3% for reoperative total arch replacement, an incidence of permanent neurological deficit of 1.4% versus 4.7%, and 10-year survival of 76% and 82.2%, respectively [51]. However, in another meta-analysis of 3154 patients undergoing elective frozen elephant trunk, operative mortality was 7.6% and stroke was 6.6% [52]. Therefore, variations in outcomes do exist in the surgical literature.

Currently, zone 0 endovascular arch intervention is largely performed for patients who are at high or prohibitive surgical risk, and therefore, direct comparisons between the surgical literature and endovascular literature have not been available. A propensity-matched study comparing open surgery to zone 0/1 TEVAR with the Japanese National Database showed that TEVAR was associated with a significantly higher incidence of stroke (5.8 vs. 10.0%, *p* < 0.001) and paraplegia/paraparesis (1.6 vs. 4.4%, *p* < 0.001), with no difference in operative mortality (4.5 vs. 5.4%) [53]. TEVAR patients were also more likely to have type 1 or 3 endoleak on postoperative imaging (6.4% vs. 1.1%). However, propensity-match cannot completely eliminate selection bias, which may significantly impact outcomes. Nonetheless, outcomes of open total arch replacement are good across high-volume aortic centers, and these outcomes would set the benchmark for endovascular aortic arch reintervention in the years to come.

## 4. Future Directions

Over the years, zone 0 endovascular arch repair had gone through multiple iterations and evolutions, and multiple investigators from around the world have advanced the field in standardizing procedural techniques and contributing to the development of available technologies. However, as a result, most studies to date for zone 0 endovascular arch repair are largely single-arm, observational studies with limited sample size, with the aim to simply report feasibility and technique. The nature of these studies limits their power for further statistical analysis to investigate potential risk factors of poor outcomes and comparison to open surgical intervention.

At the current stage, to improve upon the outcomes of zone 0 endovascular arch repair, the focus of studies should move beyond feasibility and early-outcome reporting, with the aim to establish a detailed pre-operative risk-stratification system to inform patient selection. Beyond initial screening for anatomical restrictions, patient-level factors that may increase the risk of significant peri-operative events should be identified. In addition, intraprocedural factors need to be carefully assessed to understand the impact of procedural technique on peri-procedural risk. This has been difficult thus far due to the limited number of cases performed at individual centers, advancement in technology and technique over the years, and heterogeneity in the technique used at each center. Comparison with outcomes from open surgery is also critical, as open surgery remains the gold standard for aortic arch intervention. Comparative analyses would also enable the identification of patient characteristics that may favor one approach over another. Endovascular and open arch repair should be supplementary, and a combined approach can improve quality of care and outcomes and minimize risk of complications. Eventually, should clinical equipoise be identified, a randomized controlled trial would be needed. Although issues such as small number of suitable patients, heterogeneous patient population, need for highly specialized surgical and interventional expertise, and multiple available devices may pose real-world barriers to such trials, innovative trial design such as registry-based randomization may be used to facilitate its conduct.

**Figure 1:**
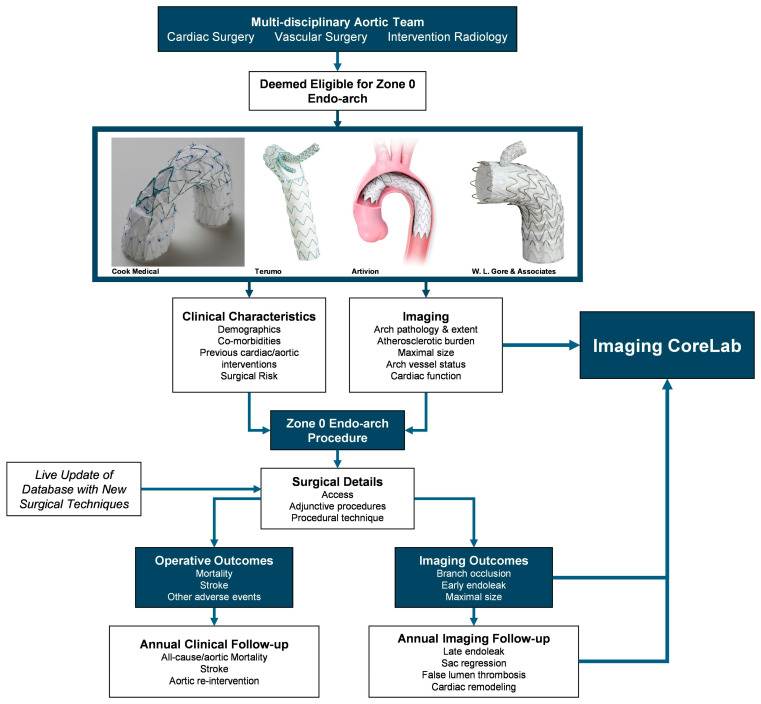
Flowchart for a proposed multicenter zone 0 endovascular arch registry *(Thoracic Ascending Branch, Courtesy of Cook Medical; Reprinted with permission of Bolton Medical, Inc. dba Terumo Aortic; Used with the Permission of Artivion, Inc.; Courtesy of W.L. Gore & Associates, Inc).*

To facilitate this, a prospective, multicenter registry from a collaborative network of expert aortic centers in open and zone 0 endovascular arch repair would be beneficial (Figure 1). A multicenter effort would ensure sufficient sample size, and a prospective nature would ensure clear data definition and standardized data collection to improve quality. In addition, standardized follow-up can be instigated to ensure collection of long-term data. One such existing network includes the United States Aortic Research Consortium, where patient outcomes for fenestrated/branched EVAR has been used to improve patient outcomes for the past decade.

Much akin to the Heart Team model employed by structural heart procedures such as transcatheter aortic valve replacement and transcatheter mitral valve intervention, complex aortic arch intervention would benefit from a multidisciplinary Aortic Team, involving cardiac surgery, vascular surgery, and interventional radiology [54]. The involvement of multiple expertise is important to ensure that patients have all treatment options available, and the ultimate therapy of choice would be determined by experts on the multidisciplinary team and in-depth informed consent to provide patients with complex arch pathology with the best treatment possible.

## 5. Conclusions

Thoracic aortic pathology involving the aortic arch can be treated with either open total arch replacement or zone 0 endovascular arch repair, with the latter currently reserved for patients with high or prohibitive surgical risk. Although endovascular arch repair has potential advantages, further studies in understanding factors that may improve short-term complications and their impact on the long-term outcomes of patients are needed. A prospective, multicenter registry from expert aortic centers with a multidisciplinary Aortic Team would facilitate further improvements in patient care in this field.

## Figures and Tables

**Table 1:** Summary of outcomes single- or multibranch devices for endovascular total arch repair.

Authors	Device	Patient No.	Age	Indication	Early Mortality	Early Stroke	Early Endoleak	Follow-up Mortality and Reintervention
**Single-Branch**								
Dake et al. [3]	TBE	8	72.8 ± 8.0	Aneurysm (100%)	0%	Disabling: 12.5% Non-disabling: 12.5%	3	1-year mortality: 2 patients Follow-up reintervention: N/A
Sweet et al. [14]	TBE	77	70.8 ± 10.8	Aneurysm (64.9%) Dissection (31.2%) Others (3.9%)	5.2%	Disabling: 7.8%	20.9%	N/A
D’Onfrio et al. [15]	NEXUS	28	72.0 ± 6.0	Aneurysm (61%) Dissection (21%) PAU (4%)	7%	Disabling: 4% Non-disabling: 3%	4%	3-year survival: 71% 3-year cumulative incidence of reintervention: 29%
Bisdas et al. [16]	NEXUS	13	71.0 ± 9.0	Aneurysm (38.5%) Dissection (38.5%) Others (23.1%)	0%	0%	N/A	1-year mortality: 2 patients
Antonello et al. [17]	NEXUS	31	73.4 ± 7.3	Aneurysm (41.9%) Dissection (41.9%) Others (16.1%)	6.5%	Major: 6.5%	19.4%	1-year survival: 83% 1-year freedom from reintervention: 97%
**Multibranch**								
Jubouri et al. [18]	Relay	108	70.7 ± 9.9	Aneurysm (75%) Dissection (25)	3.7%	Disabling: 3.7% Non-disabling: 2.8%	5.6%	1-year mortality: 4 patients 2-year freedom from reintervention: 82.4%
Singh et al. [19]	Relay	23	67.7 ± 10.6	Aneurysm (65.2%) Dissection (34.8%)	0%	0%	N/A	2-year mortality: 0% 2-year freedom from reintervention: 100%
Iglesias et al. [20]	Relay	12	74.0 ± 7.0	Aneurysm (75%) Others (25%)	8.3%	Major: 8.3%	N/A	2-year survival: 83% 15-month reintervention: 1 patient
Weijde et al. [21]	Relay	11	--	Aneurysm (81.8%) Others (18.2%)	18.2%	Disabling: 18.2% Non-disabling: 18.2%	0%	17-month mortality: 2 patients 17-month reintervention: 2 patients
Ferrer et al. [7]	Relay	7	76 (70–85)	--	28.6%	Any: 14.3%	0%	2-year mortality: 2 patients 2-year reintervention: 0%
Czerny et al. [8]	Relay	43	73.0 ± 9.0	Aneurysm (61%) Dissection (16%) Others (23%)	9%	Disabling: 7% Non-disabling: 19%	16%	16-month mortality: 11 patients 16-month reintervention: 4 patients
Kudo et al. [22]	Relay	25	81 (76–84)	Aneurysm (88%) Dissection (12%)	0%	Any: 16%	0%	1-year survival: 87.4% Follow-up reintervention: N/A
Haulon et al. [11]	Cook	38	71 (64–74)	Aneurysm (74%) Dissection (26%)	13.2%	Any: 15.8%	28.9%	1-year mortality: 4 patients 1-year reintervention: 3 patients
Tsilimparis et al. [23]	Cook	54	68 ± 10	Aneurysm (45%) Dissection (48%) Others (7%)	6%	Major: 5.5% Minor: 5.5%	N/A	1-year survival: 82% 1-year freedom from reintervention: 83%
Tenorio et al. [24]	Cook	39	70 ± 7	Aneurysm (36%) Dissection (64%)	5%	Major: 2.5% Minor: 2.5%	N/A	1-year survival: 90% 1-year freedom from reintervention: 60%
Verscheure et al. [25]	Cook	70	69 (62–74)	Dissection (100%)	2.9%	Any: 2.9%	N/A	10-month mortality: 8 patients 10-month reintervention: 20 patients
Guo et al. [12]	Cook	213	72 (65–77)	Aneurysm (39.4%) Dissection (52.1%) Others (8.5%)	8.9%	Any: 10.8%	32.9%	4-year survival: 71.8%
Nana et al. [26]	Cook	102	N/A	N/A	10.7%	Any: 13.7%	N/A	2-year survival: 79.5% 2-year freedom from reintervention: 53.9%

*N/A = not available.*

## Data Availability

No new data were created or analyzed in this study.
